# Breast Surgery Using Thoracic Paravertebral Blockade and Sedation Alone

**DOI:** 10.1155/2014/127467

**Published:** 2014-08-21

**Authors:** James Simpson, Arun Ariyarathenam, Julie Dunn, Pete Ford

**Affiliations:** ^1^Department of Anaesthesia, South Devon Healthcare NHS Foundation Trust, Torbay Hospital, Lowes Bridge, Torquay TQ2 7AA, UK; ^2^Department of Anaesthesia, Royal Devon & Exeter NHS Foundation Trust, Barrack Road, Exeter EX2 5DW, UK

## Abstract

*Introduction*. Thoracic paravertebral block (TPVB) provides superior analgesia for breast surgery when used in conjunction with general anesthesia (GA). Although TPVB and GA are often combined, for some patients GA is either contraindicated or undesirable. We present a series of 28 patients who received a TPVB with sedation alone for breast cancer surgery. *Methods*. A target controlled infusion of propofol or remifentanil was used for conscious sedation. Ultrasound guided TPVB was performed at one, two, or three thoracic levels, using up to 30 mL of local anesthetic. If required, top-up local infiltration analgesia with prilocaine 0.5% was performed by the surgeon. *Results*. Most patients were elderly with significant comorbidities and had TPVB injections at just one level (54%). Patient choice and anxiety about GA were indications for TVPB in 9 patients (32%). Prilocaine top-up was required in four (14%) cases and rescue opiate analgesia in six (21%). *Conclusions*. Based on our technique and the outcome of the 28 patients studied, TPVB with sedation and ultrasound guidance appears to be an effective and reliable form of anesthesia for breast surgery. TPVB with sedation is a useful anesthetic technique for patients in which GA is undesirable or poses an unacceptable risk.

## 1. Introduction

Acute postoperative pain occurs after breast cancer surgery in approximately 36% of patients [1] and is a key risk factor for the development of chronic pain [1, 2]. Thoracic paravertebral block (TPVB) provides superior analgesia for breast cancer surgery when used in conjunction with general anesthesia (GA) [3] and reduces the severity of chronic pain after mastectomy [4]. Although TPVB and GA are often combined [3], for some patients GA is either contraindicated or undesirable due to factors including frailty, comorbidities, anxiety and patient choice.

TPVB alone has previously been compared with GA alone [3]. However, much of the literature is heterogeneous and includes landmark techniques at multiple thoracic levels [5] which are time consuming, uncomfortable, and expose the patient to risk with each needle pass. A block from T1–T6 is required for most breast cancer surgeries. TPVB has recently undergone resurgence with improvements in ultrasound technology, affording many benefits including direct visualisation of local anesthetic (LA) spread and the pleura [3, 6]. This enables larger volumes to be injected at fewer levels whilst still achieving adequate analgesia.

We present a series of 28 patients who received an ultrasound guided TPVB with sedation alone at one, two, or three levels, for breast cancer surgery at a single UK centre between 2008 and 2012.

## 2. Methods

Patients were identified by retrospective database analysis followed by notes review for the period 2008 to 2012, at the Royal Devon and Exeter NHS Foundation Hospital, UK. Ethics committee approval was not obtained given the historical nature of the data. In all cases the same anesthesiologist (author 4) performed the block and the same surgeon (author 3) either performed or supervised the surgery.

**Figure 1:**
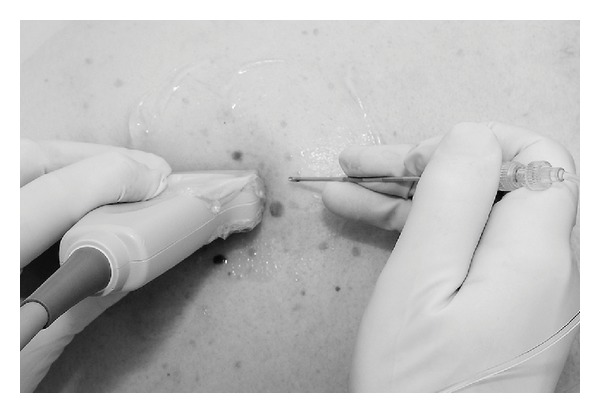
Thoracic paravertebral block technique using a 16 G Tuohy needle with a craniocaudal in-plane technique and a high frequency ultrasound transducer.

Although propofol was initially used (4/28 cases) a target controlled infusion (TCI) of remifentanil was used from 2009 onwards (24/28 cases) for conscious sedation during block placement and surgery. This was delivered using Minto's pharmacokinetic model [7] with effect site concentrations of up to 2*η*g*·*mL^−1^. A high frequency linear array ultrasound transducer and enhanced needle visualization software [8] was used, together with the Sonosite S Series Ultrasound System. A 16 G Tuohy needle (Portex, Smiths Medical, Ashford, UK) was guided to the paravertebral space with a craniocaudal in-plane technique [5] relative to the ultrasound transducer (Figure 1). LA was injected at one, two, or three thoracic levels, according to patient weight, observed spread, and operative procedure. The mean LA volume used was 24 mL (range 20–30 mL). This was initially bupivacaine 0.5% but from 2009 onwards was changed to a 50 : 50 mixture with Lignocaine 2% and Bupivacaine 0.5% to improve onset time, with the addition of Clonidine 150 *μ*g to extend the block time. Adequate block was confirmed by hypotension due to sympathetic blockade, the loss of temperature sensation over appropriate dermatomes, and by the surgeon prior to incision testing pinprick sensation in the operative area. For some cases requiring an axillary lymph node dissection, intercostobrachial nerve sacrifice was necessary for surgical reasons but the nerve itself was not infiltrated with LA. If required, top-up local infiltration analgesia into the surgical field was performed by the surgeon using prilocaine 0.5% intraoperatively. Although care was taken to ensure that toxic doses of LA were not exceeded, prilocaine 0.5% was used because of its favourable safety profile. Rescue analgesia was administered in the recovery area postoperatively if needed.

## 3. Results

28 female patients were identified during the study period, whose ASA status ranged from II to IV. Most were elderly (mean age 73, range 27–93). 24 (86%) patients had significant comorbidities which either indicated TPVB alone, or were contributory factors in the decision making process. The most frequent indications and their corresponding ages are summarised in Table 2. Three (11%) patients received TPVB due to anxiety about GA, six (21%) due to patient choice, and one (4%) who was pregnant (27 week gestation). None had previously experienced a severe adverse reaction to GA or awareness.

10 TPVB injections were performed at two levels (T3 and T5), two at three levels (T3, T5 and T6), and the remaining 16 at just one level (six at T3, eight at T4, one at T5, and one at T6). The mean total volume of LA used was 24 mL. Prilocaine 0.5% top-up was required in four (14%) cases; however, the exact sites were not recorded so it is not possible to comment on whether these may represent areas of contralateral innervation. Satisfactory operating conditions, defined as absence of pain or anything other than mild discomfort, were achieved for all except one patient. In this case, extensive comorbidities precluded GA. The operation was therefore cancelled and the patient's condition managed conservatively. There were no significant episodes of apnea during any of the cases.

Rescue opiate (tramadol, fentanyl, or morphine) analgesia was required in the recovery area in six (21%) cases. All of these had an axillary incision for either sentinel node biopsy (SNB, 4/6) or axillary lymph node dissection (ALND, 2/6). Two patients required antiemetics in the recovery area postoperatively. One had a wide local excision with TPVB performed at one level with no additional opiates required. The other had a mastectomy and SNB with TPVB at three levels but also required fentanyl 75 mcg postoperatively which may have been a contributory factor to her nausea. Results including surgical case breakdown and length of stay data are summarised in Table 1.

## 4. Discussion

Anesthesia for this group of patients can be challenging due to comorbidities, frailty, advancing age, and anxiety. Whilst these are not absolute indications for TPVB with sedation rather than GA, many are relative contraindications to GA and were therefore contributory factors in the decision making process following full discussions with the patients beforehand. Providing satisfactory operating conditions without GA whilst maintaining patient confidence, comfort, and dignity is potentially a problem, but our cohort demonstrates that it is possible with ongoing refinement of the anesthetic technique. This is highlighted by the case of one 57-year-old lady who required a second procedure, electing again to have it performed under TPVB with sedation.

Of central importance to our technique is the close working relationship between anesthesiologist and surgeon. This is key not only for intraoperative issues such as the occasional need for LA top-up, but also to introduce TPVB with sedation as an option at an early stage for suitable patients. For many of our cases it was first discussed as a possibility in the outpatient clinic by the surgeon. The early administration of sedation in the anesthetic room was also crucial, as TPVB can be uncomfortable during the injection of LA as the pleura is displaced ventrally. Although a TCI of propofol was initially used, in our experience it sometimes results in confusion and disinhibition. From 2009 onwards, the patients with remifentanil conscious sedation were more tolerant, conversant, and cooperative. Its potent analgesic properties and favourable pharmacokinetics were also advantageous with the time pressures on a busy list, where a solo anesthesiologist is required to deliver operating conditions in a timely fashion. It therefore helped with list efficiency, as did the 50 : 50 mixture of lignocaine 2% and bupivacaine 0.5% which improved onset time, rather than pure bupivacaine 0.5%. Clonidine 150 *μ*g was also added to this mixture to extend the block duration, although the extra time gained is likely to have been modest [9]. Block durations were not formally measured but were generally in the order of 24 hours, with no significant differences between the two different LA mixtures used. The use of clonidine and remifentanil probably contributed to the need for vasoactive drugs (typically ephedrine in the range of 6–12 mg intraoperatively) due to sympathectomy, but there were no problems with postoperative hypotension or cardiovascular instability. We did not notice a difference in rescue opioids between patients sedated with propofol and those sedated with remifentanil.

The choice of paravertebral level was made on a case by case basis, taking into account operative site, quality of ultrasound view, and observed spread of LA during injection. For example, in cases requiring multiple dermatomal levels of anesthesia (such as mastectomy with ALND), one level of injection was only performed if good spread of LA was seen craniocaudally with real-time ultrasound visualization. This general approach to limit the number of needle passes aimed to minimise patient discomfort, complication rate, and anesthetic time. Although postoperative chest X-rays (CXR) looking for pneumothorax were not routinely performed, there were no patients in whom respiratory compromise was evident postoperatively and therefore no CXRs were performed.

The most significant shortcoming of our technique was the requirement for opiates in the recovery area in six cases. Although no record of the exact indication was available, all were patients in whom an axillary lymph node dissection or sentinel node biopsy had been performed. This reflects the challenge of regional anesthesia in an area which is innervated by a number of different nerves. The Pecs II block [10] has been described as a rescue or additional technique in breast surgery and may provide a useful alternative, providing analgesia to the axillary region and pectoral muscles which are largely innervated by the brachial plexus. Those patients having a wide local excision, wire localized biopsy, or cavity reexcision required little or no top-up infiltration analgesia or opiates in recovery. Whilst these groups may be managed with local infiltration analgesia and sedation or GA, in our experience they are sometimes disproportionately painful resulting in significant rescue opiate administration and unplanned admissions.

Our typical length of stay (LOS) following mastectomy is one day, whilst less invasive procedures such as wide local excision and wire localized biopsy are often done as day cases. Mean LOS data is summarized in Table 1, including individual reasons for particular delays which have skewed the mean values. Other common reasons for delays were social issues, physiotherapy, and rehabilitation, reflecting the predominantly elderly and often frail population studied.

## 5. Conclusions

Based on our technique and the outcome of the 28 patients studied, TPVB with sedation and ultrasound guidance appears to be an effective and reliable form of anesthesia for breast surgery. Those requiring a sentinel node biopsy or axillary lymph node dissection are most likely to require extra analgesia postoperatively and in these patients additional regional techniques may be of benefit. TPVB with sedation is a useful anesthetic technique for patients in which GA is undesirable or poses an unacceptable risk.

## Figures and Tables

**Table 1:** Summary table of surgical cases, number of dermatomal levels blocked, prilocaine 0.5% top-up, intercostobrachial (ICB) nerve sacrifice, recovery opiate requirement, and length of stay data.

Procedure TPVB levels	*n*	Mean LOS (days)	Prilocaine top-up (*n*)	ICB Nerve cut (*n*)	Recovery opiates (*n*)	Comments
Mastectomy						
1 level	4	2.0	2	0	0	Longest LOS 4 days, social reasons
Mastectomy with ANC						
1 level	3	3.0	1	2	0	Longest LOS 5 days, social reasons
2 levels	3	1.0	0	3	1	
Mastectomy with SNB						
1 level	1	1.0	0	0	0	
2 levels	2	2.0	1	0	2	
3 levels	2	4.5	0	0	1	Longest LOS 7 days, infective diarrhoea
Wide local excision						
1 level	3	0	0	0	0	
Wide local excision with SNB						
1 level	2	0	0	0	1	
2 levels	3	1.3	0	0	0	
Wire localised biopsy						
1 level	2	0	0	0	0	
2 levels	1	0	0	0	0	
Cavity re-excision						
1 level	2	0.5	0	0	1	
Total	**28**		**4**	**5**	**6**	

**Table 2:** Indications for TPVB alone and corresponding ages (years). Indications are not mutually exclusive (i.e., individual patients may have more than one).

Indication	*n*	Age, mean (range)
Anxiety/patient choice		
Significant anxiety/phobia of GA	3	66.0 (57–84)
Stated patient choice	6	58.8 (27–85)
Pregnancy (27-week gestation)	1	27 (27-27)
Comorbidities		
Hypertension	16	82.8 (58–90)
Heart failure	4	88.0 (85–89)
Ischaemic heart disease	5	72.6 (53–90)
Valvular heart disease	4	74.3 (64–92)
Stroke/transient ischaemic attack	10	69.7 (53–89)
Significant respiratory disease	5	71.2 (64–85)
Chronic kidney disease	6	79.8 (53–92)
Spinal abnormalities/difficult airway	2	57.5 (57-58)
Chronic pain	2	57.0 (57-57)
